# Dissecting Adult Peripheral Cellular Immunity to *Bordetella pertussis*: Innate-Associated IFN-γ Responses, Transient T-Cell Recall, and HLA-A*02-Associated CD8+ Reactivity

**DOI:** 10.3390/cells15191807

**Published:** 2026-10-03

**Authors:** Alexandra Maleeva, Varvara Shokina, Zlata Naydenysheva, Anna Lyapina, Eleonora Sadykova

**Affiliations:** Research Institute for Systems Biology and Medicine (RISBM), Scientific Driveway, 18, Moscow 117246, Russia

**Keywords:** *Bordetella pertussis*, cellular immunity, IFN-γ, T-cell recall, CD4+ T cells, CD8+ T cells, HLA-A*02, ELISpot, innate immunity

## Abstract

**Highlights:**

- Peripheral IFN-γ responses to *B. pertussis* comprise distinct innate-associated and T-cell-associated components that can be resolved by spot-pattern ELISpot analysis and stimulation context.
- Circulating *B. pertussis*-reactive T-cell responses contract rapidly after clinical pertussis, while detectable CD8+ IFN-γ reactivity is preferentially associated with HLA-A*02 positivity.
- Peripheral IFN-γ measurements should be interpreted according to cellular composition and stimulation kinetics, as short-term PBMC responses may not selectively reflect antigen-specific T-cell recall to pertussis.
- Time since infection and HLA class I genotype should be considered when assessing human cellular immunity to *B. pertussis* and designing pertussis immunomonitoring studies.

**Abstract:**

*Bordetella pertussis* elicits both innate and adaptive cellular responses, yet their relative contributions to peripheral interferon-γ (IFN-γ) measurements and the temporal dynamics of circulating T-cell reactivity in adults remain incompletely defined. *B. pertussis*-reactive cellular immunity was investigated in 42 adult donors, including 26 healthy donors and 16 donors with a history of pertussis, using standard and modified IFN-γ enzyme-linked immunospot (ELISpot) assays with a chemically inactivated whole-cell *B. pertussis* preparation, bacterial innate-biased stimuli, and a peptide-based T-cell recall control, together with human leukocyte antigen A*02 (HLA-A*02) genotyping. Short-term stimulation of unfractionated peripheral blood mononuclear cells (PBMCs) generated readily detectable IFN-γ spot formation that was not clearly stratified by childhood vaccination background or previous pertussis history. In a subset of seven donors, lipopolysaccharide and *B. pertussis*-derived lipooligosaccharide generated strong total PBMC signals but little detectable pattern-defined T-cell-associated spot formation, whereas the whole-cell pertussis preparation retained a detectable T-cell-associated component under modified assay conditions. Among seven donors available for longitudinal follow-up after pertussis, pattern-defined T-cell-associated IFN-γ spot frequencies in peripheral blood were highest at the recovery sampling point, were significantly reduced at one month, decreased further by three months, remained markedly reduced at six months, and approached background levels at 12 months after recovery. Directly measured CD8+ IFN-γ responses were preferentially detected in HLA-A*02-positive donors. These findings indicate that cellular composition and sampling time affect the interpretation of peripheral IFN-γ responses to *B. pertussis* and show an association between HLA-A*02 status and detectable CD8+ reactivity.

## 1. Introduction

Whooping cough (pertussis) is a respiratory infection caused by *Bordetella pertussis* and remains an important global public-health problem despite widespread vaccination [1,2,3]. Although antibodies contribute to protection against symptomatic disease, cellular immunity is increasingly recognized as an important determinant of long-term protection [2,4,5,6,7]. In particular, CD4+ T cells are known to be critical for clearance of *B. pertussis* infection [2,8,9], and both natural infection and vaccination generate complex *B. pertussis*-reactive T-cell populations.

Although *B. pertussis* has traditionally been regarded as a predominantly extracellular respiratory pathogen, this view has been refined by experimental evidence showing that the bacterium can enter and persist within host phagocytic cells, including human macrophages [10,11,12,13]. These observations do not imply an obligate intracellular lifestyle; instead, they but broaden the immunological framework of pertussis beyond exclusively extracellular antibacterial mechanisms. Accordingly, although studies of adaptive cellular immunity to *B. pertussis* have focused predominantly on the CD4+ T-cell compartment [3,7,14,15,16], a potential contribution of CD8+ T cells should not be excluded. Natural infection and whole-cell pertussis vaccination are associated predominantly with Th1/Th17-type responses, whereas the phenotype of vaccine-induced immunity is strongly influenced by the original vaccination background [6,7,14,15]. Previous human studies have also demonstrated that both CD4+ and CD8+ lymphocytes can contribute to antigen-induced IFN-γ production in response to *B. pertussis* antigens [16].

Because antigen recognition by CD8+ T cells depends on peptide presentation by HLA class I molecules, HLA genotype represents a potential source of inter-individual heterogeneity in detectable CD8+ T-cell responses. HLA-dependent differences in peptide presentation may influence the breadth and magnitude of infection- or vaccine-induced CD8+ T-cell reactivity and are therefore relevant to the interpretation of cellular immunomonitoring; however, a direct relationship between HLA-A*02 status and protection against pertussis has not been established. HLA class I restriction of human *B. pertussis*-specific responses remains poorly characterized. Among the limited available epitope data, HLA-A*02:01 binding has been reported for a *B. pertussis*-derived peptide [17], providing a rationale for examining whether detectable CD8+ IFN-γ reactivity was associated with HLA-A*02 status.

IFN-γ is an important functional mediator and readout of type 1 cellular immunity to *B. pertussis* [12]. Measurement of antigen-induced IFN-γ production is therefore widely used to assess cellular reactivity after infection or vaccination, and IFN-γ enzyme-linked immunospot (ELISpot) assays enable sensitive enumeration of cytokine-producing cells [18]. However, an IFN-γ ELISpot signal does not itself identify either the phenotype or the antigen specificity of the responding cell. This limitation is particularly important when unfractionated peripheral blood mononuclear cells (PBMCs) are stimulated with complex bacterial preparations. *B. pertussis* can activate innate immune pathways, leading to pronounced IFN-γ production, including macrophage-dependent activation of natural killer (NK) cells [19,20]. Consequently, short-term stimulation of unfractionated PBMCs may generate an integrated IFN-γ signal derived from several cellular populations rather than selectively measuring antigen-specific T-cell recall. Strong early innate-associated IFN-γ production may therefore obscure less abundant *B. pertussis*-reactive T-cell responses. Distinguishing these components is essential for the interpretation of IFN-γ-based immunomonitoring of cellular immunity to *B. pertussis*.

The present study was therefore designed to distinguish rapid mixed-cell IFN-γ responses from T-cell-associated reactivity to *B. pertussis* in adult peripheral blood. First, short-term IFN-γ responses to a chemically inactivated whole-cell *B. pertussis* preparation were characterized in unfractionated PBMCs. Second, responses to whole-cell *B. pertussis* were compared with bacterial innate-biased stimuli and a peptide-based T-cell recall control under standard and modified stimulation conditions designed to improve discrimination between the early mixed-cell IFN-γ signal and the T-cell-associated component. The temporal dynamics of circulating *B. pertussis*-reactive T-cell responses were subsequently examined after recent pertussis, together with the separate contributions of CD4+ and CD8+ T-cell populations. Finally, the association between detectable CD8+ IFN-γ reactivity and HLA-A*02 status was assessed.

## 2. Materials and Methods

### 2.1. Donors

Peripheral venous blood was obtained from 42 adult donors (19–54 years), including 26 healthy donors (23–54 years) and 16 donors recovered from pertussis (19–34 years). All participants provided written informed consent in accordance with the Declaration of Helsinki, under a study protocol (protocol No. 1, approved 28.05.2024) approved by the Institutional Ethics Committee of the Research Institute for Systems Biology and Medicine (RISBM).

The study population comprised 23 women and 19 men. The healthy donor group included 14 women and 12 men, whereas the recovered group included 9 women and 7 men (Table 1).

Sex distribution did not differ significantly between healthy and recovered donors (Fisher’s exact test, *p* = 1.000). Age distributions differed significantly between the groups (two-sided Mann–Whitney U test, *p* = 0.009), with a median age of 32.0 years in healthy donors and 25.5 years in recovered donors.

Information on childhood pertussis vaccination status and history of pertussis during childhood was obtained from medical records. Exact dates of individual pertussis vaccine doses, including the most recent vaccination, were not extracted. Healthy donors had no recent clinically recognized pertussis or symptoms of infectious disease at enrollment and were further characterized according to childhood vaccination background and remote pertussis history. Donors in the recovered group had a history of a medically certified sick-leave episode diagnosed as pertussis.

In the longitudinal follow-up, 14 donors were included: seven healthy donors (26–41 years) and seven donors who had recovered from pertussis (24–35 years). The longitudinal healthy subgroup comprised five women and two men. These donors underwent serial blood sampling at baseline and subsequently at approximately one, three, six, 12, 16, and 25 months in the absence of clinically apparent pertussis during the observation period. The longitudinal recovered subgroup comprised three women and four men. For these seven participants, official documentation of the pertussis-associated sick-leave period was available.

All longitudinally followed recovered donors had positive *B. pertussis*-specific IgG and IgA serological results reported by the respective diagnostic laboratories at variable time points relative to the acute illness and sick-leave period. Information on the specific serological assays, quantitative antibody values, and laboratory-specific diagnostic cut-offs was not collected. PCR testing for *B. pertussis* was additionally performed in 3/7 longitudinally followed recovered donors and was positive in all three. All longitudinally followed recovered donors reported receiving antibiotic therapy during the acute illness; information on the specific antimicrobial agents, doses, and treatment duration was not collected.

For longitudinal analyses of recovered donors, “recovery” was operationally defined as the first day following closure of the medical sick-leave period. The exact interval between symptom onset and initiation of medical sick leave was not available.

Blood samples from recovered donors were obtained at the defined recovery time point and subsequently at approximately one month, three months, six months, and 12 months after recovery.

For the matched standard-versus-modified ELISpot comparison, seven donors were included, comprising five healthy donors and two recovered donors sampled one month after recovery. The subset size and composition reflected the donors from whom blood was collected at the time this methodological experiment was performed. This methodological subset was distinct from the seven recovered donors included in the longitudinal post-pertussis analysis.

### 2.2. HLA-A*02 Genotyping

HLA-A*02 status was determined using two sources of HLA typing data. Seven of the 42 donors had pre-existing HLA genotyping that had been performed by next-generation sequencing on an Illumina platform and provided for the study. These pre-existing HLA typing results had been generated independently before enrollment; detailed information on the library-preparation protocol, HLA-calling software, and analysis pipeline was not available for the present study.

For the remaining 35 donors, HLA-A*02 status was determined in-house using an HLA-A*02-specific polymerase chain reaction with sequence-specific primers (PCR-SSP) adapted from the method described by Liang et al. [21].

Genomic DNA was isolated from peripheral whole blood using the HiPure Universal DNA Kit (cat. IVD3018, Magen Biotechnology, Guangzhou, China) according to the manufacturer’s instructions.

The HLA-A*02-specific reaction used the forward primer 5′-TCCTCGTCCCCAGGCTCT-3′ and reverse primer 5′-GTGGCCCCTGGTACCCGT-3′, generating an expected 813-bp HLA-A*02-specific amplicon. An independent internal amplification control targeting the adenomatous polyposis coli (APC) gene was performed using primers 5′-ATGATGTTGACCTTTCCAGGG-3′ and 5′-ATTCTGTAACTTTTCATCAGTTGC-3′, generating a 256-bp control product. The HLA-A*02-specific and internal-control amplifications were performed in separate reactions. Each reaction was carried out in a total volume of 25 μL containing 150 ng of genomic DNA, 0.5 μL of 10 mM deoxynucleoside triphosphate (dNTP) mix (cat. PB006L, Eurogen), 2.5 μL of 10× Taq polymerase buffer (cat. PK017S, Eurogen), 0.25 μL of Taq DNA polymerase (cat. PK017S, Eurogen), and 1 μL of the primer mix containing 10 μM of each primer. For the HLA-A*02-specific reaction, amplification consisted of an initial denaturation at 96 °C for 5 min; five cycles of 96 °C for 35 s, 70 °C for 55 s, and 72 °C for 55 s; 21 cycles of 96 °C for 35 s, 65 °C for 55 s, and 72 °C for 55 s; and then 10 cycles of 96 °C for 35 s, 70 °C for 55 s, and 72 °C for 55 s, with a final extension at 72 °C for 10 min. The internal-control reaction was performed using an initial denaturation at 96 °C for 5 min, followed by 36 cycles of 96 °C for 35 s, 63.5 °C for 55 s, and 72 °C for 55 s, with a final extension at 72 °C for 10 min.

PCR products were analyzed by agarose gel electrophoresis. Samples showing the expected 813-bp HLA-A*02-specific product were classified as HLA-A*02-positive. Samples lacking the 813 bp product were classified as HLA-A*02-negative only when successful amplification of the 256 bp internal-control product confirmed a valid PCR reaction.

To ensure comparability between the two typing approaches, all donors were analyzed at the binary HLA-A*02-status level—individuals carrying at least one HLA-A*02 allele were classified as HLA-A*02-positive, whereas all others were classified as HLA-A*02-negative.

Exact HLA-A*02 subtype determination was not required for the present analysis.

### 2.3. Isolation of Peripheral Blood Mononuclear Cells and CD4+ and CD8+ T Cells

Peripheral blood mononuclear cells (PBMCs) were isolated from peripheral venous blood collected into heparinized tubes by Ficoll density-gradient centrifugation (cat. P050E, PanEco, Moscow, Russia), as previously described [22]. PBMCs were isolated immediately after blood collection and used fresh on the same day for all experiments.

PBMCs were used either as unfractionated cell populations or as the starting material for subsequent isolation of CD4+ and CD8+ T-cell fractions.

For experiments requiring direct assessment of CD4+ and CD8+ T-cell responses, PBMCs were separated by magnetic cell isolation. CD8+ T cells were isolated using CD8 MicroBeads, human (cat. 130-045-201, Miltenyi Biotec, Bergisch Gladbach, Germany), and CD4+ T cells using CD4 MicroBeads, human (cat. 130-045-101, Miltenyi Biotec, Germany), according to the manufacturer’s instructions after stimulation with the corresponding stimulus in bulk culture (described below). The purity of the isolated CD4+ and CD8+ T-cell fractions was >93%, as determined by flow-cytometry validation.

### 2.4. Standard and Modified IFN-γ Enzyme-Linked Immunospot (ELISpot) Assay

For the standard ELISpot assay, freshly isolated PBMCs were plated directly onto ready-to-use Human IFN-γ ELISpot Plus (horseradish peroxidase, HRP) plates (cat. 3420-4HST-10, Mabtech, Nacka Strand, Sweden) in CTL-Test Medium (cat. CTLT-010, Cellular Technology Limited, Shaker Heights, OH, USA) supplemented with 50 µg/mL gentamicin (cat. 1.3.16, BioloT, Saint-Petersburg, Russia). Cells were stimulated for 18–24 h with a chemically inactivated whole-cell *B. pertussis* preparation (FSO.3.2.00089, Scientific Centre for Expert Evaluation of Medicinal Products of Ministry of Health of the Russian Federation, Moscow, Russian Federation) that was used as the whole-cell *B. pertussis* stimulus throughout the study at a final concentration of 3 × 10^7^ bacterial cells per well. The preparation (FSO.3.2.00089) was supplied as a chemically inactivated whole-cell *B. pertussis* reference preparation. The available documentation did not specify the *B. pertussis* strain designation or the detailed chemical inactivation procedure.

For the modified ELISpot assay, PBMCs were first incubated with the corresponding stimulus for 12 h in bulk culture and were subsequently transferred to IFN-γ ELISpot plates for an additional 24 h incubation under the same stimulation condition. The 12 h pre-incubation step was introduced to facilitate discrimination between early mixed-cell IFN-γ production and the T-cell-associated component detected during subsequent ELISpot analysis.

For each sample, unstimulated wells were included as negative controls. The corresponding background SFC count was subtracted from the stimulated-well SFC count before normalization and statistical analysis. Cells stimulated with phytohemagglutinin (PHA; cat. A023, PanEco, Russia) at final concentration 5 µg/mL were used as a positive control.

For the matched stimulus-comparison experiments PBMCs were analyzed under both the standard and modified assay conditions with the following stimuli: the whole-cell *B. pertussis* preparation (described above) as the primary complex bacterial test stimulus; lipopolysaccharide (LPS; cat. GC205009-10mg, Wuhan Servicebio Technology Co., Wuhan, China) at a final concentration of 0.1 µg/mL and *B. pertussis*-derived lipooligosaccharide (LOS, archived laboratory stock) at a final concentration of 0.1 µg/mL as innate-biased comparator stimuli; and cytomegalovirus (CMV) pp65 peptide set at a final concentration of 0.6 µM of each peptide (PepTivator CMV pp65, Miltenyi Biotec, Germany) as a peptide-based T-cell recall control.

Cells were plated in twofold serial dilutions, typically starting at 5 × 10^5^ cells per well and decreasing to approximately 1 × 10^5^ cells per well. When cell yield was limited, lower starting cell numbers were used while maintaining the twofold serial-dilution scheme.

Following incubation, ELISpot plates were developed according to the manufacturer’s instructions and acquired using an ImmunoSpot S6 Ultra M2 analyzer (Cellular Technology Limited, Shaker Heights, OH, USA). Spots were counted using ImmunoSpot software (v.5.0) with manually optimized counting protocols. A spot was defined as a spatially discrete IFN-γ signal identified by the software and accepted by the manually optimized counting protocol; each accepted spot was counted as one spot-forming cell (SFC).

To establish reference patterns for T-cell-associated IFN-γ spots, magnetically purified CD4+ and CD8+ T-cell fractions were analyzed as internal cellular reference populations. Spot-morphology parameters were calibrated using these reference wells, and the resulting settings were subsequently applied to unfractionated PBMC datasets to identify pattern-defined T-cell-associated SFCs. Spot counts were reported as IFN-γ SFCs per 10^6^ PBMC equivalents.

### 2.5. Statistical Analysis

Each donor was treated as an independent biological replicate. Sex distributions between healthy and recovered donors were compared using Fisher’s exact test, and age distributions were compared using the two-sided Mann–Whitney U test. For comparisons between two independent groups, the two-sided Mann–Whitney U test was used. Cross-sectional comparisons among three or four independent groups were performed using Kruskal–Wallis test, followed by Dunn’s multiple-comparisons test. For complete longitudinal datasets containing repeated measurements from the same donors and for matched comparisons of multiple stimulation conditions within the same donors, the Friedman test, followed by Dunn’s multiple-comparisons test, was applied. For longitudinal datasets containing missing observations, a mixed-effects model fitted by restricted maximum likelihood (REML), followed by Šídák’s multiple-comparisons test, was used. When multiple Mann–Whitney comparisons were performed within the same set of comparisons, *p*-values were adjusted using the Holm–Bonferroni method.

Cross-sectional and matched-stimulus data are presented as individual donor values with the median and interquartile range (IQR), whereas longitudinal data are presented as individual donor trajectories. A two-sided *p*-value < 0.05 was considered statistically significant. Statistical significance is indicated as follows: ns, not significant; * *p* < 0.05; ** *p* < 0.01; *** *p* < 0.001; and **** *p* < 0.0001.

Statistical analysis was performed using GraphPad Prism v.8 (GraphPad Software, Boston, MA, USA).

## 3. Results

### 3.1. Rapid B. pertussis-Induced IFN-γ Responses in PBMCs Are Not Clearly Stratified by Previous Immune History

Under the standard short-term IFN-γ ELISpot assay (Figure 1A), stimulation of unfractionated PBMCs with the chemically inactivated whole-cell *B. pertussis* induced readily detectable IFN-γ spot formation in adult donors. In the cross-sectional analysis, response magnitude did not differ significantly among donors who had recovered from pertussis in adulthood, vaccinated donors with a history of pertussis during childhood, and vaccinated donors without a known history of pertussis (Figure 1B). This cross-sectional comparison was independent of the longitudinal analysis of recently recovered donors shown in Figure 1D. Thus, under the standard short-term assay conditions, pertussis history and childhood vaccination background did not clearly stratify the magnitude of the total PBMC IFN-γ response.

In healthy donors followed longitudinally (n = 7), short-term PBMC IFN-γ responses showed intra-individual variability across serial measurements from baseline through 25 months of follow-up, without a consistent directional change in the absence of clinically apparent pertussis (Figure 1C). In donors followed longitudinally after recovery from pertussis (n = 7), responses were highest at the recovery point, decreased by approximately one month, and showed a pronounced further reduction by approximately three months, remaining lower at later follow-up time points (Figure 1D).

The cellular source of the total IFN-γ signal detected in the standard 18–24 h assay cannot be assigned directly from these data. However, the presence of a readily detectable IFN-γ signal within this relatively shorter stimulation interval, together with previous reports demonstrating *B. pertussis*-induced IFN-γ production by NK cells [19,20], is consistent with a contribution from innate or innate-like cells. Accordingly, the total IFN-γ signal measured in unfractionated PBMCs under the standard assay conditions should not be interpreted exclusively as a quantitative measure of *B. pertussis*-specific memory T-cell recall.

### 3.2. Bacterial Innate-Biased Stimuli and Antigenic Recall Generate Distinct Total PBMC and Pattern-Defined T-Cell-Associated IFN-γ Response Patterns

A matched methodological comparison was performed to evaluate whether the modified *B. pertussis* stimulation protocol could distinguish the pattern-defined T-cell-associated component of the whole-cell *B. pertussis* response from responses elicited by bacterial innate-biased comparator stimuli and a peptide-based T-cell recall control. A subset of seven donors, comprising five healthy donors and two recovered donors sampled one month after recovery, was included in this analysis. Matched PBMC samples from each donor were tested under four stimulation conditions—chemically inactivated whole-cell *B. pertussis*, *B. pertussis*-derived LOS, LPS, and a CMV pp65 peptide set—using both the standard and modified ELISpot formats (Figure 2). The standard format consisted of direct 18–24 h ELISpot stimulation, whereas the modified format included a 12 h bulk pre-incubation with the corresponding stimulus, followed by a 24 h ELISpot incubation under the same stimulation condition.

Under standard assay conditions, readily detectable total IFN-γ responses were induced by LOS and LPS in unfractionated PBMCs, and both responses exceeded that observed following whole-cell *B. pertussis* stimulation (Figure 2B). The largest total PBMC response among the tested stimuli was observed following stimulation with the CMV pp65 peptide set. When the same ELISpot wells were re-analyzed using pattern-based counting, a different response profile was obtained (Figure 2C). Little or no pattern-defined T-cell-associated IFN-γ reactivity was detected following LOS or LPS stimulation, and these responses did not differ significantly from that observed following whole-cell *B. pertussis* stimulation. A pronounced pattern-defined T-cell-associated IFN-γ response was detected following CMV pp65 stimulation. Thus, the strong LOS- and LPS-associated total PBMC signals were not accompanied by correspondingly strong pattern-defined T-cell-associated responses.

Under the modified assay conditions, a readily detectable total PBMC IFN-γ response was observed following whole-cell *B. pertussis* stimulation (Figure 2D). Within this assay format, the response to whole-cell *B. pertussis* was significantly greater than that induced by LOS, whereas no significant differences were detected between whole-cell *B. pertussis* and either LPS or CMV pp65.

In the pattern-defined T-cell-associated readout under the modified conditions, a clearer separation among the bacterial stimuli was observed (Figure 2E). One possible explanation is that rapidly responding innate or innate-like populations and T-cell-associated recall differ in their temporal contribution to the IFN-γ signal across the two assay configurations; however, the present experiments were not designed to determine the underlying cellular kinetics directly. A readily detectable pattern-defined T-cell-associated IFN-γ response was observed following whole-cell *B. pertussis* stimulation and was significantly greater than the responses induced by either LOS or LPS, both of which remained close to background levels. Strong pattern-defined T-cell-associated reactivity was retained following CMV pp65 stimulation and exceeded that observed following whole-cell *B. pertussis* stimulation. In Figure 2C,E, the pattern-defined readout was treated as a combined T-cell-associated signal and was not resolved into separate CD4+ and CD8+ T-cell subsets. Direct measurements of magnetically separated CD4+ and CD8+ T-cell fractions are presented in Figure 3D–F.

### 3.3. Circulating Pattern-Defined T-Cell-Associated IFN-γ Responses Show Temporal Variability and Rapid Post-Infection Contraction

The magnitude and longitudinal behavior of the circulating pattern-defined T-cell-associated IFN-γ response to *B. pertussis* were subsequently characterized by re-analysis of the ELISpot data using the calibrated T-cell-associated spot-pattern criteria. As in Figure 1B, Figure 3A represents the cross-sectional donor-group comparison and is distinct from the longitudinal post-pertussis analysis shown in Figure 3C.

In cross-sectional analysis, response magnitude of the pattern-defined T-cell-associated IFN-γ response did not differ significantly among donors who had recovered from pertussis in adulthood, vaccinated donors with a history of pertussis during childhood, and vaccinated donors without a known history of pertussis (Figure 3A). Thus, no clear stratification of the circulating response according to remote pertussis history or childhood vaccination background was observed at the time of sampling.

Considerable intra-individual variation over time was observed in the longitudinal analysis of healthy donors (n = 7; Figure 3B). No consistent directional change was detected from baseline through approximately one, three, six, 12, 16, and 25 months of follow-up, indicating that the magnitude of the detectable circulating pattern-defined T-cell-associated response can vary considerably over time even in the absence of a recent clinically recognized infection.

A different temporal pattern was observed in donors followed longitudinally after recovery from pertussis (n = 7; Figure 3C). Pattern-defined T-cell-associated IFN-γ responses were highest at recovery and declined progressively during follow-up. A significant reduction was already detectable approximately one month after recovery, followed by a further pronounced decrease by approximately three months. Responses remained markedly reduced at six months and approached background levels in most donors at 12 months after recovery.

Figure 1B–D and Figure 3A–C represent different analytical readouts of ELISpot responses obtained from the same donor samples. In Figure 1B–D, all IFN-γ spots meeting the general ELISpot counting criteria are included in the total PBMC SFC count. In Figure 3A–C, the same ELISpot responses are re-analyzed using spot-morphology parameters calibrated on magnetically purified CD4+ and CD8+ T-cell reference wells, thereby identifying the pattern-defined T-cell-associated component. Consequently, the lower absolute SFC values in Figure 3A–C reflect the more restrictive pattern-based analytical definition rather than differences in donor composition or sampling time.

Overall, a pronounced but transient increase in the circulating pattern-defined T-cell-associated IFN-γ response was observed following recent pertussis, followed by rapid contraction over the subsequent months. This contraction reflects the dynamics of the circulating compartment and should not be interpreted as evidence of complete loss of *B. pertussis*-specific cellular memory. In contrast, no stable stratification of the peripheral response according to remote pertussis history or childhood vaccination background was observed.

### 3.4. B. pertussis-Reactive CD8+ IFN-γ Responses Are Associated with HLA-A*02

To determine the relative contributions of CD4+ and CD8+ T-cell compartments to the recall response, IFN-γ responses were measured in magnetically separated CD4+ and CD8+ T cells following bulk PBMC stimulation with the chemically inactivated whole-cell *B. pertussis* preparation (Figure 3D–F).

CD4+ IFN-γ responses were readily detectable in both healthy and recovered donors, with no significant difference between these groups (Figure 3D). CD8+ responses were lower and more heterogeneous, with several donors showing little or no detectable activity.

In contrast, detectable CD8+ IFN-γ responses were preferentially observed in HLA-A*02-positive donors (Figure 3E) and were significantly greater than in HLA-A*02-negative donors, in whom responses were predominantly absent or close to background. The same association was observed when healthy and recovered donors were analyzed separately (Figure 3F). Within both groups, CD8+ IFN-γ responses were greater among HLA-A*02-positive individuals, whereas HLA-A*02-negative donors showed little detectable CD8+ activity.

These findings indicate an association between HLA-A*02 status and the detectability of the circulating *B. pertussis*-reactive CD8+ IFN-γ response. However, direct HLA-A*02 restriction at the epitope level was not demonstrated by these experiments.

## 4. Discussion

Several cellularly and temporally distinct features of adult peripheral IFN-γ reactivity to *B. pertussis* were identified. First, short-term stimulation of unfractionated PBMCs with the whole-cell *B. pertussis* preparation generated a readily detectable total IFN-γ response that was not clearly stratified by childhood vaccination background or remote pertussis history. Second, strong total PBMC responses to LOS and LPS were accompanied by little detectable pattern-defined T-cell-associated reactivity, whereas a strong pattern-defined response was retained following CMV pp65 stimulation, and a detectable T-cell-associated component was observed following whole-cell *B. pertussis* stimulation. Third, the circulating pattern-defined T-cell-associated IFN-γ response was highest at recovery from pertussis and contracted rapidly during the subsequent months. Finally, directly measured CD8+ IFN-γ responses were preferentially detected in HLA-A*02-positive donors.

The distinction between the total PBMC and pattern-defined T-cell-associated readouts is important for the interpretation of short-term IFN-γ measurements. Strong total PBMC responses were observed following LOS and LPS stimulation, whereas little pattern-defined T-cell-associated reactivity was detected under the same stimulation conditions. This pattern is consistent with a contribution from non-T-cell populations and with previous studies demonstrating macrophage-dependent activation of IFN-γ-producing NK cells by *B. pertussis* [19,20]. However, neither NK cells nor other innate or innate-like populations were directly identified as the source of individual ELISpot signals in the present experiments. Accordingly, the total IFN-γ signal detected after short-term stimulation of unfractionated PBMCs should not be interpreted exclusively as a quantitative measure of *B. pertussis*-specific memory T-cell recall.

The CMV pp65 peptide set provided an important peptide-based T-cell recall control. Pronounced pattern-defined T-cell-associated reactivity was detected following CMV pp65 stimulation, indicating that the low LOS- and LPS-associated pattern-defined signals were not attributable to a general inability to detect T-cell-associated IFN-γ responses. A different response profile was observed following stimulation with the whole-cell *B. pertussis* preparation: in addition to the total PBMC response, a detectable pattern-defined T-cell-associated component was observed, particularly under the modified assay conditions. The 12 h pre-incubation step was introduced to facilitate discrimination between early mixed-cell IFN-γ production and the T-cell-associated component detected during subsequent ELISpot analysis. In the matched comparison performed in the present study, a clearer separation of whole-cell *B. pertussis* from the innate-biased comparator stimuli was observed in the pattern-defined readout under the modified conditions. However, this methodological comparison was not designed as a formal analytical validation of assay sensitivity or reproducibility.

An important limitation of IFN-γ ELISpot interpretation is also illustrated by these observations. Although IFN-γ ELISpot provides sensitive detection of cytokine-secreting cells [18], IFN-γ secretion alone does not establish either the cellular phenotype or the antigen specificity of the responding cell. This distinction is particularly relevant when complex bacterial preparations containing both antigenic proteins and innate immune stimuli are used. Cellular context, stimulation conditions, and the analytical definition of the measured response should therefore be considered when interpreting *B. pertussis*-induced IFN-γ reactivity.

A further finding was the rapid temporal contraction of the circulating pattern-defined T-cell-associated IFN-γ response after pertussis. Responses were highest at the recovery sampling point, decreased during the first one to three months, and approached background levels in most donors at 12 months after recovery. Previous studies have similarly shown that both time since infection and age can influence the magnitude and persistence of circulating *B. pertussis*-specific cellular responses [23]. Importantly, reduced reactivity in peripheral blood should not be interpreted as complete disappearance of cellular immunological memory. *B. pertussis*-reactive respiratory tissue-resident CD4+ T cells producing IFN-γ and IL-17 have been reported to persist for decades [5]. Because only peripheral blood was examined in the present study, the persistence or distribution of tissue-resident memory could not be assessed directly. The observed decline therefore describes the circulating compartment and is compatible with previous evidence that peripheral and respiratory tissue memory may display different temporal dynamics.

The absence of clear differences in circulating IFN-γ response magnitude according to remote pertussis history or childhood vaccination background should also be interpreted within the limits of the measured readout. Long-lasting effects of childhood vaccination background on the qualitative phenotype of *B. pertussis*-specific CD4+ T-cell responses have been demonstrated in previous human studies [6,7,14,15]. Therefore, the absence of clear differences in IFN-γ SFC magnitude in the present study does not exclude vaccination-dependent qualitative immune imprinting.

An association between directly measured CD8+ IFN-γ reactivity and HLA-A*02 status was also observed. Human cellular immunity to *B. pertussis* has historically been characterized predominantly within the CD4+ T-cell compartment, although both CD4+ and CD8+ lymphocytes have been shown to contribute to antigen-induced IFN-γ production [16]. The preferential detection of CD8+ responses in HLA-A*02-positive donors is consistent with a contribution of HLA class I genotype to inter-individual variation in the magnitude or detectability of circulating *B. pertussis*-reactive CD8+ responses. This association should not, however, be interpreted as direct evidence of HLA-A*02 restriction. A complex whole-cell bacterial preparation was used, and the peptides recognized by responding CD8+ T cells were not identified. Demonstration of HLA restriction would require peptide-specific stimulation, HLA-blocking experiments, peptide–MHC multimer analysis, or epitope mapping. Similarly, IFN-γ production demonstrates functional activation but does not establish a cytotoxic phenotype; direct assessment of cytotoxic mediators or target-cell killing would be required.

Several limitations should be considered. The study population was modest in size, particularly for the matched standard-versus-modified assay comparison (n = 7) and the HLA-stratified analyses, thus limiting the precision of subgroup comparisons. Healthy donors and donors with a history of pertussis differed significantly in age distribution, with recovered donors being younger; therefore, an influence of age on between-group comparisons cannot be excluded. Sex distributions were similar descriptively, but sex-specific effects were not evaluated. Although all recovered donors had a medically certified sick-leave episode diagnosed as pertussis, laboratory documentation was heterogeneous and did not constitute a uniform laboratory case definition across the full recovered cohort. Detailed serological information was systematically available for the seven longitudinally followed recovered donors, whereas PCR testing was performed in only three of these seven donors. In addition, the exact relationship between symptom onset and initiation of medical sick leave was unavailable. Exact dates of individual childhood pertussis vaccine doses, including the most recent vaccination, were not extracted.

The experimental approach also imposes several limitations. Only peripheral blood was examined, and IFN-γ was used as a single functional readout; consequently, tissue-resident immunity, broader T-cell functional profiles, cytotoxicity, and protective capacity were not assessed. Pattern-defined T-cell-associated SFCs were classified according to spot morphology calibrated using magnetically purified CD4+ and CD8+ reference wells rather than by direct cellular phenotyping within the same PBMC samples. Furthermore, use of a chemically inactivated whole-cell *B. pertussis* preparation does not permit attribution of the observed responses to individual bacterial antigens or epitopes. These limitations are particularly relevant to interpretation of the HLA-A*02 association, and conclusions regarding direct peptide–HLA restriction cannot be drawn from the present data.

## 5. Conclusions

In summary, adult peripheral IFN-γ reactivity to *B. pertussis* was resolved into experimentally distinguishable total PBMC and T-cell-associated components. Strong short-term IFN-γ responses were detected in unfractionated PBMCs, whereas pattern-defined T-cell-associated reactivity showed distinct stimulus-dependent profiles and was more clearly resolved under the modified assay conditions. Circulating pattern-defined T-cell-associated IFN-γ responses were highest at recovery from pertussis and contracted markedly during subsequent follow-up, approaching background levels in most donors at 12 months after recovery. Directly measured CD8+ IFN-γ responses were preferentially detected in HLA-A*02-positive donors, indicating an association with HLA-A*02 status without demonstrating direct HLA-A*02 restriction. Accordingly, cellular context, assay configuration, sampling time, and host HLA class I background should be considered when interpreting peripheral IFN-γ responses to *B. pertussis*.

## Figures and Tables

**Figure 1:**
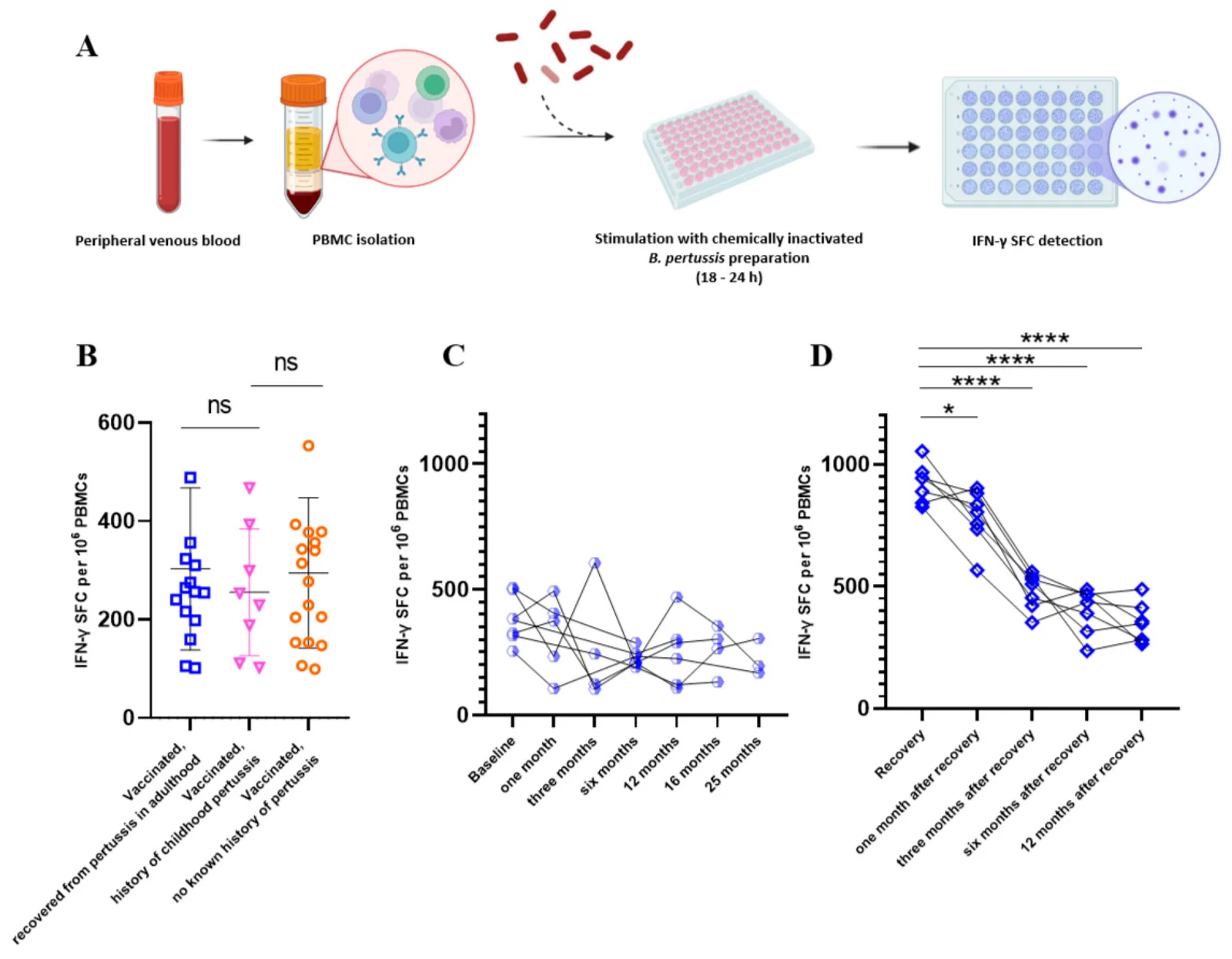
Short-term IFN-γ responses to *B. pertussis* in peripheral blood of healthy and recovered donors. (**A**) Experimental design of the short-term IFN-γ ELISpot assay. Freshly isolated PBMCs were stimulated with chemically inactivated whole-cell *B. pertussis* preparation for 18–24 h before IFN-γ SFC detection. (**B**) IFN-γ responses in adult donors (n = 42) stratified according to pertussis history and childhood vaccination background. Individual symbols represent individual donors; data are presented as median with interquartile range (IQR). Kruskal–Wallis test followed by Dunn’s multiple-comparisons test. (**C**) Longitudinal variation in short-term PBMC IFN-γ responses in healthy donors (n = 7), assessed at baseline and approximately one, three, six, 12, 16, and 25 months thereafter. Individual lines represent individual donors. Mixed-effects model (REML) followed by Šídák’s multiple-comparisons test. (**D**) Longitudinal kinetics of short-term PBMC IFN-γ responses in donors recovered from pertussis (n = 7), assessed at recovery and approximately one, three, six, and 12 months after recovery. Individual lines represent individual donors. Friedman test followed by Dunn’s multiple-comparisons test. A two-sided *p*-value < 0.05 was considered statistically significant. *ns*, not significant; * *p* < 0.05; **** *p* < 0.0001.

**Figure 2:**
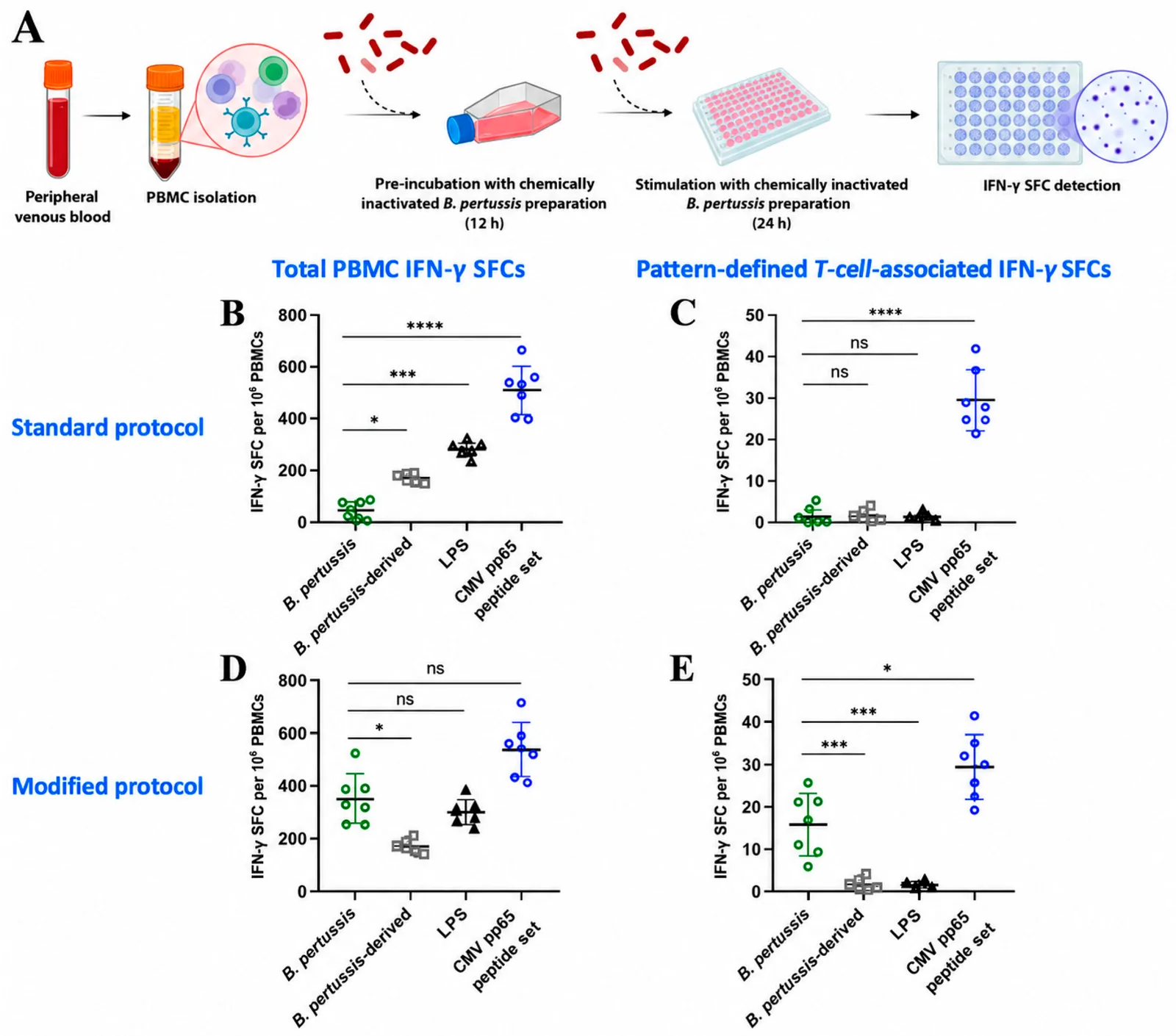
IFN-γ response patterns to whole-cell *B. pertussis*, bacterial innate-biased stimuli, and peptide-mediated T-cell recall under standard and modified ELISpot conditions. (**A**) Experimental workflow of the modified ELISpot assay. Freshly isolated PBMCs underwent a 12 h pre-incubation step before subsequent 24 h ELISpot analysis; the standard protocol consisted of direct ELISpot stimulation without the 12 h pre-incubation step. (**A**) illustrates the modified assay workflow using the whole-cell *B. pertussis* preparation as the primary test stimulus. LOS, LPS, and the CMV pp65 peptide set were used in parallel comparator conditions to evaluate the ability of the modified assay to distinguish the *B. pertussis*-associated T-cell readout from innate-biased and peptide-mediated responses. (**B**) Total IFN-γ SFC responses in unfractionated PBMCs under the standard protocol conditions. (**C**) Pattern-defined T-cell-associated IFN-γ SFCs identified in unfractionated PBMCs under the standard protocol conditions. (**D**) Total IFN-γ SFC responses in unfractionated PBMCs under the modified protocol conditions. (**E**) Pattern-defined T-cell-associated IFN-γ SFCs identified in unfractionated PBMCs under the modified protocol conditions. The T-cell-associated readout in (**C**,**E**) was configured by spot-pattern analysis calibrated using magnetically purified CD4+ and CD8+ T-cell reference wells and therefore represents a combined pattern-defined T-cell-associated readout. A subset of donors (n = 7, five healthy and two recovered donors at one month after recovery) was included in this methodological comparison. The same PBMC sample from each donor was analyzed across all four stimuli and both assay formats; cells were stimulated with the chemically inactivated whole-cell *B. pertussis*, *B. pertussis*-derived LOS and LPS as innate-biased comparator stimuli, or a CMV pp65 peptide set as a peptide-based T-cell recall. Individual donor values are shown with the median and interquartile range (IQR). Within each experimental condition and readout, differences among matched stimuli were analyzed using the Friedman test followed by Dunn’s multiple-comparisons test, with whole-cell *B. pertussis* used as the reference condition. *ns*, not significant; * *p* < 0.05; *** *p* < 0.001; **** *p* < 0.0001.

**Figure 3:**
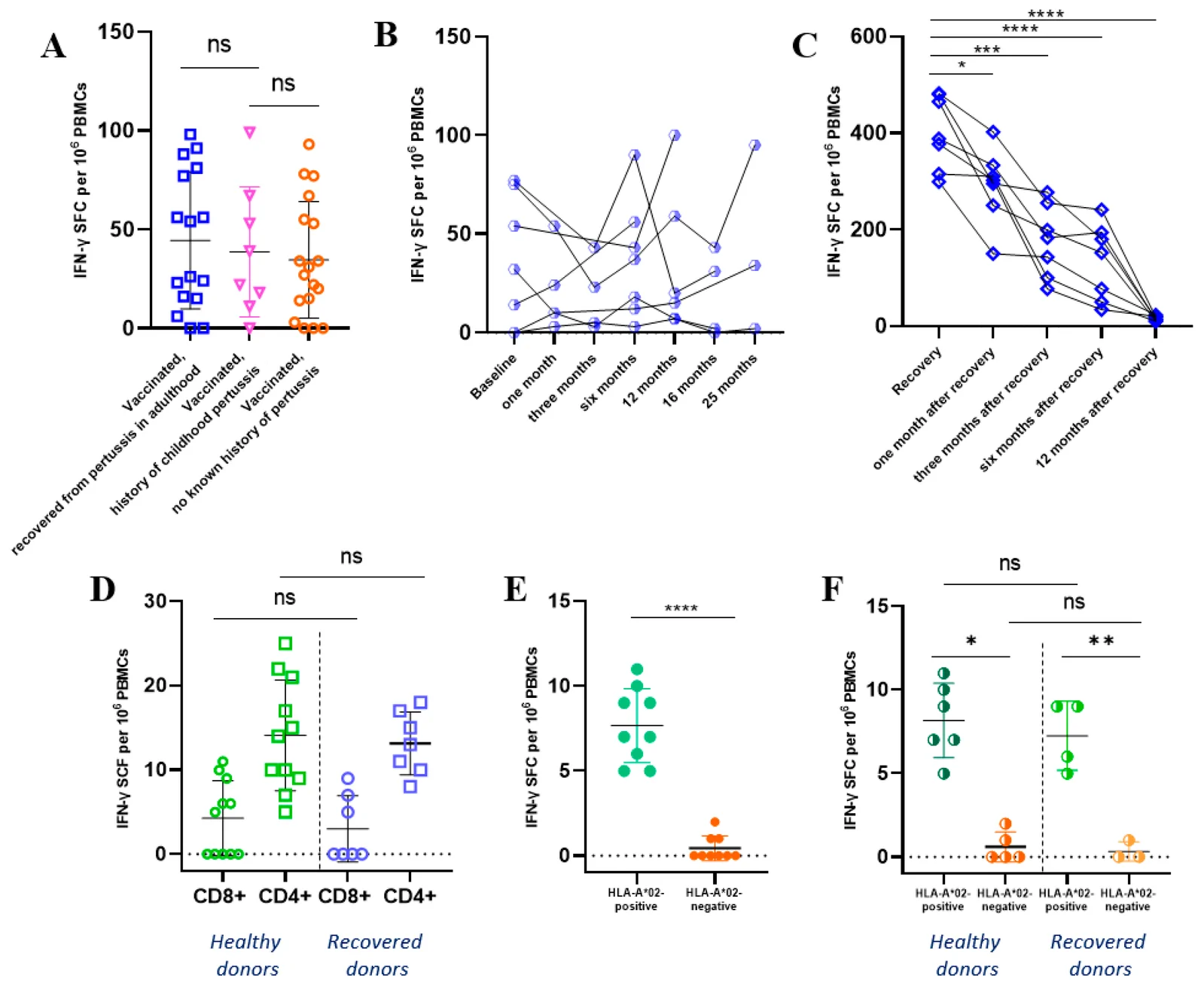
Temporal dynamics and cellular composition of circulating *B. pertussis*-reactive IFN-γ responses. (**A**) Pattern-defined T-cell-associated IFN-γ SFC responses in adult donors stratified according to pertussis history and childhood vaccination background. Kruskal–Wallis test followed by Dunn’s multiple-comparisons test. (**B**) Longitudinal variation in pattern-defined T-cell-associated IFN-γ responses in healthy donors (n = 7) at baseline and approximately one, three, six, 12, 16, and 25 months thereafter. Mixed-effects model (REML) followed by Šídák’s multiple-comparisons test. (**C**) Longitudinal kinetics of pattern-defined T-cell-associated IFN-γ responses in donors recovered from pertussis (n = 7), assessed at recovery and approximately one, three, six, and 12 months after recovery. Friedman test followed by Dunn’s multiple-comparisons test. Panels (**A**–**C**) show the T-cell-associated component identified by spot-pattern analysis in unfractionated PBMCs. (**D**) Direct IFN-γ responses of magnetically separated CD8+ and CD4+ T-cell fractions in healthy and recovered donors. Mann–Whitney U test with Holm–Bonferroni correction for multiple comparisons. (**E**) Direct CD8+ IFN-γ responses according to HLA-A*02 status across donors. Mann–Whitney U test. (**F**) Direct CD8+ IFN-γ responses according to HLA-A*02 status within healthy and recovered groups. Mann–Whitney U test with Holm–Bonferroni correction for multiple comparisons. Individual symbols represent individual donors. Cross-sectional data in (A, D–F) are presented as median with interquartile range (IQR); longitudinal plots in (**B**,**C**) show individual donor trajectories. A two-sided *p*-value < 0.05 was considered statistically significant. *ns*, not significant; * *p* < 0.05; ** *p* < 0.01; *** *p* < 0.001; **** *p* < 0.0001.

**Table 1:** Demographic and clinical characteristics of the study participants.

Characteristic	All Donors	Healthy Donors	Recovered Donors
n	42	26	16
Age (range)	19–54	23–54	19–34
Women, n (%)	23 (54.8%)	14 (53.8%)	9 (56.3%)
Men, n (%)	19 (45.2%)	12 (46.2%)	7 (43.8%)
*Longitudinal follow-up,* *n/N*	14/42	7/26	7/16
*Women, n (%)*	*8/14 (57.1%)*	*5/7 (71.4%)*	*3/7 (42.9%)*
*Men, n (%)*	*6/14 (42.9%)*	*2/7 (28.6%)*	*4/7 (57.1%)*
*Age (range)*	*24–41*	*26–41*	*24–35*
Positive *B. pertussis*-specific IgG and IgA ^1^, n/N with available data	-	-	7/7
Positive *B. pertussis* PCR ^1^, n/N tested	-	-	3/3
Antibiotic treatment during acute illness ^2^, n/N with available data	-	-	7/7

^1^ Clinical laboratory and treatment information was systematically available for recovered donors included in longitudinal follow-up. ^2^ Antibiotic treatment was reported by the participants; the specific antimicrobial agents, doses, and treatment duration were not collected.

## Data Availability

The original contributions presented in this study are included in the article material. Further inquiries can be directed to the corresponding author.

## Abbreviations

The following abbreviations are used in this manuscript:

CD4+	Cluster of Differentiation 4-positive T cells
CD8+	Cluster of Differentiation 8-positive T cells
CMV	Cytomegalovirus
ELISpot	Enzyme-linked immunospot
HLA	Human Leukocyte Antigen
HLA-A*02	Human Leukocyte Antigen A*02
HRP	Horseradish peroxidase
IFN-γ	Interferon gamma
IgA	Immunoglobulin A
IgG	Immunoglobulin G
IQR	Interquartile range
LOS	Lipooligosaccharide
LPS	Lipopolysaccharide
MHC	Major Histocompatibility Complex
NGS	Next-generation sequencing
NK	Natural killer cell
PBMCs	Peripheral blood mononuclear cells
PCR	Polymerase chain reaction
PHA	Phytohemagglutinin
REML	Restricted maximum likelihood
SFCs	Spot-forming cells
